# A computational approach to “free will” constrained by the games we play

**DOI:** 10.3389/fnint.2012.00085

**Published:** 2012-09-27

**Authors:** Kenneth T. Kishida

**Affiliations:** Computational Psychiatry Unit and Human Neuroimaging Laboratory, Virginia Tech Carilion Research Institute, Virginia TechRoanoke, VA, USA

**Keywords:** free will, human decision-making, dopamine, neuroeconomics, computational psychiatry, fMRI, electrochemistry, computational reinforcement learning theory

## Abstract

Human choice is not free—we are bounded by a multitude of biological constraints. Yet, within the various landscapes we face, we do express choice, preference, and varying degrees of so-called willful behavior. Moreover, it appears that the capacity for choice in humans is variable. Empirical studies aimed at investigating the experience of “free will” will benefit from theoretical disciplines that constrain the language used to frame the relevant issues. The combination of game theory and computational reinforcement learning theory with empirical methods is already beginning to provide valuable insight into the biological variables underlying capacity for choice in humans and how things may go awry in individuals with brain disorders. These disciplines operate within abstract quantitative landscapes, but have successfully been applied to investigate strategic and adaptive human choice guided by formal notions of optimal behavior. Psychiatric illness is an extreme, but interesting arena for studying human capacity for choice. The experiences and behaviors of patients suggest these individuals fundamentally suffer from a diminished capacity of willful choice. Herein, I will briefly discuss recent applications of computationally guided approaches to human choice behavior and the underlying neurobiology. These approaches can be integrated into empirical investigation at multiple temporal scales of analysis including the growing body of experiments in human functional magnetic resonance imaging (fMRI), and newly emerging sub-second electrochemical and electrophysiological measurements in the human brain. These cross-disciplinary approaches hold promise for revealing the underlying neurobiological mechanisms for the variety of choice capacity in humans.

## Introduction

A scientific perspective of “free will” must be rooted in the parts of the problem that are measureable and consistent with the wealth of empirical data. Others have discussed the need for this kind of approach and make good philosophical arguments for the progress a scientific framework promises (Dennett, 2003; Churchland and Churchland, 2006; Suhler and Churchland, 2009). But, before we can address the neurodynamics of “free will” the question, “*What is* free will?” must be addressed. Unfortunately an empirical answer to even this obvious question remains murky—narratives aimed at describing the subjective experiences associated with free will fail to pin down concrete variables for measurement and experimentation. However, discussions on the topic of free will lurk around issues related to observable choice behavior and the causes of our actions. In the extreme, no organism has *complete* freedom in their capacity to make choices. There are great numbers of evolved physical constraints that restrict any given organism's capacity to choose; for example, humans cannot *choose* to breath underwater (unassisted) no more than a fish can *choose* to become a doctor. Yet, we observe choice behavior all around us and this phenomenon is a very active area of research for many academic disciplines including neuroscience, economics, psychology, politics, and philosophy.

Choice behavior is arguably the basis for all that is interesting in humans. The impact of our choices pervades all aspects of our day-to-day existence and has long-term consequences for our planet and the life it supports. The choices individuals make are likely the basis for how we assign personality and define identity. The apparent freedom individuals possess in carrying out these decisions is a highly valued concept in many cultures. The social value of this concept and beliefs about “free will” may have evolved around the complex social development of our species. Thus, we hold others (and ourselves) accountable for our actions; and, we reward or punish individuals based on our acceptance of the choices expressed. Accountability and the choices we make in social domains assume that we possess agency and determine the course of our own actions (Skyrms, 1996). In cases where agency appears to be diminished (e.g., mental illness) we debate the accountability of individuals that have performed unacceptable acts. This suggests that altered brain chemistry or abnormal brain development are examples where individuals' freedom to choose are accepted to be diminished. Still, this intuition lacks from a rigorous framework that could guide empirical determinations of humans' capacity for choice. Such a framework will be important to guide what it is we mean when we discuss “free will.”

Mathematically explicit models have been incorporated into empirical investigations of choice behavior and are beginning to generate good hypotheses about how nervous systems deal with computations associated with various choice problems. Herein, I will briefly introduce two dominant quantitative theories that deal with choice behavior (i.e., game theory and computational reinforcement learning theory). I will discuss the impact these disciplines have already had on neurobiological investigations of human choice behavior and highlight how the integration of these disciplines is beginning to open new arenas for investigating the biological basis for choice capacity. These arenas include the diminished capacity for choice experienced by individuals with mental illness and investigation into the variation expressed by healthy decision makers. It is in the intersection of theoretical and empirical disciplines where advances in our understanding of our capacities for choice can develop. I will conclude with a depiction of possible future avenues of basic neuroscience research in the domain of human choice capacity and how these investigations may impact psychiatric medicine and more generally our conception of “free will.”

## Integrating theoretical disciplines with an empirical neuroscience of choice

A neurobiological understanding into the mechanisms underlying willful choice behavior in humans must integrate the wide range of knowledge gained from empirical studies at multiple scales of analysis. Molecular systems build neural circuits that respond to and direct the actions of a wholly integrated organism; the behavior of these systems can be measured in a wide range of spatial and temporal scales, thus complicating the job of any integrative hypothesis. Quantitative theory has had a big impact on integrating the growing body of neurobiological data, but much work remains (Abbott, 2008). Investigations into the neurobiology underlying choice behavior has recently been infused with the quantitative theory of games (Montague and Berns, 2002; Montague et al., 2006; Camerer, 2008). The establishment of game theory (Von Neumann and Morgenstern, 1947) provided an abstract, but principled and mathematical approach to the problem of choice during strategic interaction. The extension of this framework to an experimental context gave birth to behavioral economics (Camerer, 2003), which recently extended its empirical investigations with human neuroimaging technology (Montague and Berns, 2002; Montague et al., 2006; Camerer, 2008; Kishida et al., 2010). Prior to the development of neuroeconomics, computational reinforcement learning theory (Sutton and Barto, 1998) provided an abstract depiction of value guided choice behavior with notions of optimally adaptive actions. Game theoretic and computational reinforcement learning approaches stand to provide a foundation for empirical investigations into choice behavior and the expression of choice capacity. In line with the aims of this special issue, these theoretical frameworks have already provided insight into the neurobiology underlying choice behavior crossing multiple temporal scales of investigation including millisecond spike activity of individual neurons (Montague et al., 1996), sub-second changes in extracellular dopamine (Clark et al., 2010; Kishida et al., 2011), to blood-oxygen level-dependent neuroimaging in humans (Berns et al., 2001; Pagnoni et al., 2002; McClure et al., 2003, 2004; D'Ardenne et al., 2008).

## Computational reinforcement learning theory

**Figure 1:**
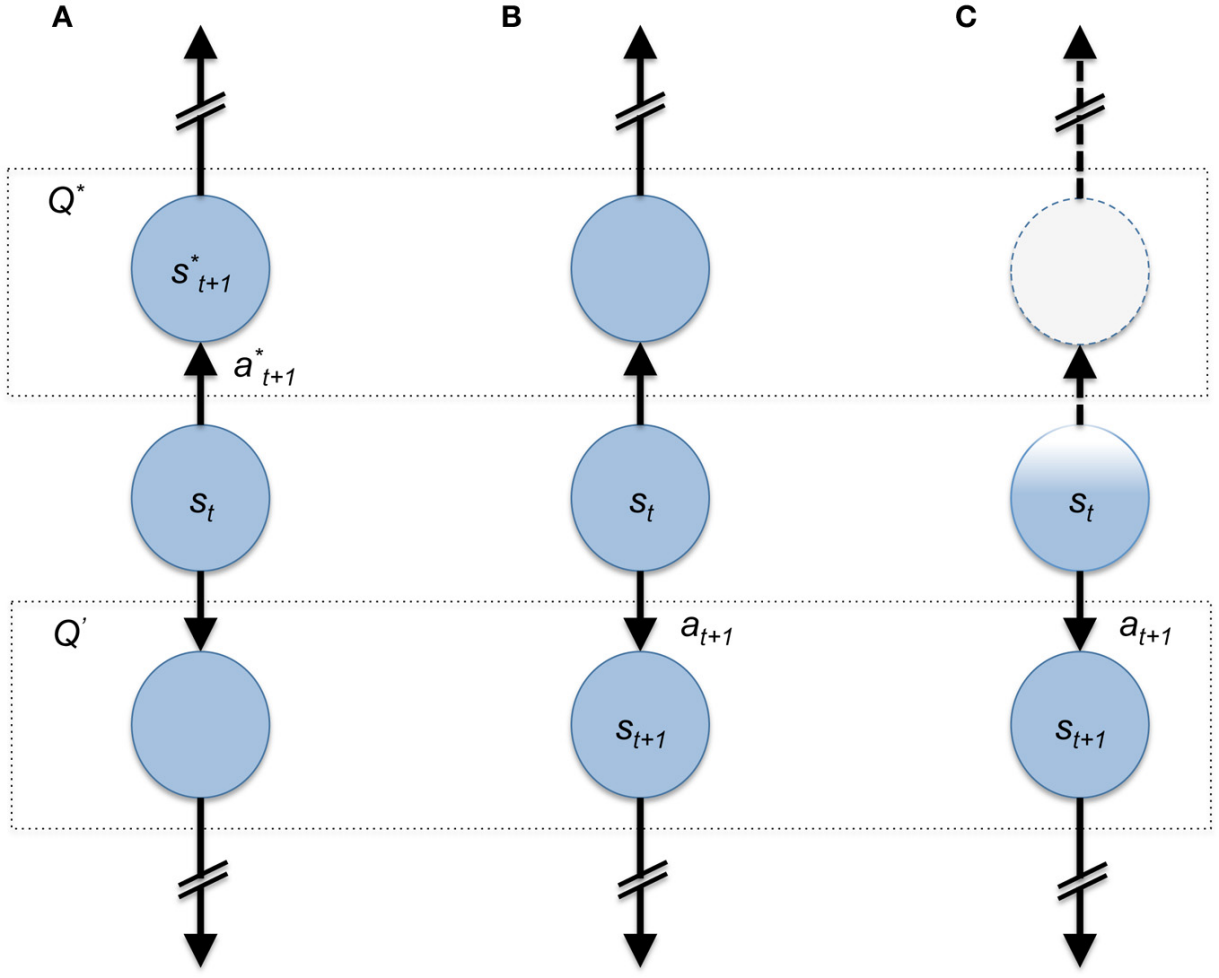
**Cartoon depiction of state-space representation of a simple choice problem.** In all three panels an agent is in state *s*_*t*_ at time *t* and must choose up or down. The dashed boxes with Q^*^ and *Q*′ represents the value of the up-state (value-maximizing) and down state (sub-optimal), respectively. In **(A)** the agent chooses a^*^_t+1_ and moves into the value maximizing (Q^*^) state s^*^_t+1_. **(B)** The agent chooses sub-optimally due to a faulty estimate of the value maximizing choice. **(C)** The agent has an incomplete representation of its current state resulting in the diminished representation of available options; here the sub-optimal choice appears to be the only one possible.

Computational reinforcement learning theory (Sutton and Barto, 1998) has relatively few fundamental moving parts. Abstractly, these include the decision-making agent, its environment (the “state-space”), guidance signals (“rewards”), and a policy. In this framework, an agent receives signals from its environment, which tells the agent all it needs to know about the current decision-problem or what state, “*s*_*t*_”, the agent is in at time “*t*” (Figure 1). As the agent traverses different states of the environment it receives new information about the new states it visits including positive or negative feedback in the form of rewards and punishments, respectively. Here, rewards are a quantitative variable determined by the environment that immediately signals to the agent “this state is good” by amount “*r*_*t*_”. With this information the agent makes a decision to take some action “*a*_*t*_” according to the agent's policy. Within the development of computational reinforcement learning theory all of these processes are captured and represented mathematically. A fundamental principle that has emerged from this theory is the Bellman optimality equations, which prescribe the optimal values to states and actions according to some policy and the expected accumulation of rewards for future states. For example:

$$Q^{*}(s,\;a)=E\left\{r_{t+1}+\gamma \underset{a^{\prime }}{\max}(Q^{*}(s_{t+1},\;a^{\prime }))|s_{t}=s,\;a_{t}=a\right\}$$

is the Bellman optimal action-value equation. It determines the optimal action-value assignment (i.e., Q^*^ (*s*, *a*)) for a given state-action pair [an action (*a*) taken while in state (*s*)]. Here “*E*” is the expectation of the reward “*r*_*t*+1_” plus the discounted (γ) state-action value function (Q^*^(*s*_*t*+1_, *a*′)) for the next state (*s*_*t*+1_) given some chosen action (*a*′); *a*′ is chosen to maximize Q^*^(*s*_*t*+1_, *a*′), for which the policy is implied. Finally, the expected value “*E*{·|(*s*_*t*_ = *s*, *a*_*t*_ = *a*)}” is calculated given the actual state-action pair at time *t* (*s*_*t*_ = *s*, *a*_*t*_ = *a*). When everything is known about the decision problem (e.g., the current state, next state, the possible actions, and their associated rewards) the calculation of this problem is straightforward. But, in the real world these decision problems are faced with incomplete knowledge and the theoretical work in this domain has been aimed at different solutions to variations on this problem. This framework has been deployed on a number of biological decision-making problems including those that humans face or are pitted against in experiments aimed at understanding how humans make decisions in environments with various statistical properties (Montague et al., 2006; Kishida and Montague, 2012; Lee and Jung, 2012).

An additional value of this framework, in the present discussion, is that it lays down the fundamental moving parts of the problem of willful choice. Within this framework we can begin to discuss concretely the ways in which an agent's choice capacity may be constrained or freed. For example, Figure 1A demonstrates the ideal case where an agent is sitting in a state and takes an accurate value maximizing action. If an agent possessed complete knowledge of the decision problem as the agent in Figure 1A is assumed, then to choose up is the only decision that satisfies the assumption that decision-makers act to maximize their return. Additionally Figures 1B,C highlight examples where an agent may make the “wrong” decision, but for different reasons: Figure 1B shows the agent choosing downward due to an inaccurate estimate of the value maximizing action. This is very likely to happen when the estimate of Q^*^ (*s*, *a*) is wrong due to incomplete or inaccurate information about the decision to be made (for example: a poor model or less than optimal learning expressed by the agent). An example of this kind of problem during willful decision making has been explored in a formal manner by Dayan et al.; here, the authors present a computational theoretical depiction of the problem faced by an agent that utilizes both reflexive and contemplative mechanisms for navigating choice spaces and show how one or the other system may misestimate the value of available options (Daw et al., 2005; Dayan et al., 2006).

Figure 1C, on the other hand, highlights a very different issue. Here, the agent does not represent or encode the state that they are in accurately (expressed by the faded color of the state circle, “*s*_*t*_,” Figure 1C). This agent also chooses down incorrectly due to the fact that the representation that “up is an option” is completely lacking because the present state is not encoded correctly. Each of these examples shows how a decision-making agent may express variable behavior dependent on the ability to represent the decision problem accurately.

## Game theory

The establishment of game theory (Von Neumann and Morgenstern, 1947) provided a quantitative framework for exploring the theory of strategic decision-making in (rational self-interested) humans. The thought experiments and the corresponding solutions developed within this discipline provided a natural entry point for quantitative experimental approaches (Camerer, 2003); and, for understanding constrained choice behavior in range of biological agents (Smith, 1982). A fundamental principle underlying game theoretic depictions of choice behavior is the notion that decision-making agents act to maximize some utility function. Decisions in this context are made rationally and to maximize the agent's selfish interests. Experimental economics and the more recent development of pairing behavioral experiments with neuroscientific measures [i.e., neuroeconomics, (Montague and Berns, 2002; Glimcher and Rustichini, 2004)] aims to test hypotheses generated within economic theory. However, the quantitative nature of game theoretic probes may prove more broadly beneficial in disciplines aimed and generally understanding the biology of choice behavior. These probes have been designed with explicit notions of optimality that ought to determine behavior. The control signals implicit in these quantitative games are ripe for experiments aimed at determining biological correlates of choice capacity. Interestingly humans often deviate from the “rational” game theoretic solution and express a range of variable responses, which are largely uncharacterized from an empirical standpoint (Camerer, 2003). This suggests that humans are not constrained by the assumptions laid down in economic theory, but these games provide abstract quantitative landscapes, which experimenters can exploit for testing decision-making models in human participants. These games have been employed in humans and non-human model organisms to test the assumptions present in economic theory and to determine the neurobiological substrates for the kinds of computations required to navigate these game spaces.

## Decision neuroscience and computational psychiatry

Decision neuroscience has been greatly influenced by psychology, computational theory, and more recently game theory. Experimental paradigms employing a strictly quantitative framework for examining choice pair naturally with the physiological measurements neuroscientists prefer. Prior to functional magnetic resonance imaging (fMRI) the primary tools available to investigate the underlying neurobiology of choice were highly invasive, and thus restricted primarily to non-human decision-making experiments. fMRI has opened the door to determining physiological responses associated with willful choice. Experiments aimed at understanding human decision-making have taken advantage of the theoretical frameworks developed in game theory and computational reinforcement learning theory (Montague et al., 2004, 2006; Kishida et al., 2010; Kishida and Montague, 2012; Lee and Jung, 2012). An exciting development along these lines is the advent of Computational Psychiatry (Kishida et al., 2010; Maia and Frank, 2011; Montague et al., 2011).

In the context of willful choice, psychiatric disorders pose a number of interesting issues. The altered behavioral profiles of patients with psychiatric disorders compared to healthy individuals suggest an amplification of the kinds of biological determinants that naturally constrain human choice. Computational psychiatry aims to characterize psychiatric illness in objectively measureable quantitative terms. Using game theoretic paradigms to investigate altered social behavior in patients diagnosed with mental disorders (Kishida et al., 2010; Maia and Frank, 2011; Montague et al., 2011) the goal of this newly inspired effort will be to determine previously hidden characterizations of the “computational” problems expressed by individuals diagnosed with mental illness [for an introduction to Computational Psychiatry please see (Montague et al., 2011)]. The early developments of computational psychiatry have focused on examining human decision-making behavior through the lens of computational reinforcement learning theory and game theoretic probes of choice behavior. These behavioral probes have been paired primarily with fMRI, but more recently, invasive measurements in humans have begun to verify and challenge some of the hypotheses generated in the theory guided fMRI experiments.

## Human electrochemistry and electrophysiology

The computational role of reward and valuation in adaptive decision-making has been explored at the level of individual neuron activity. In 1996 Montague et al., proposed a mathematical model of dopamine neuron activity in non-human primates [(Montague et al., 1996) and reviewed in Schultz et al. (1997)]. These models later guided valuation experiments in humans using fMRI. Initially these experiments investigated simple reward and valuation responses (Berns et al., 2001; Pagnoni et al., 2002; McClure et al., 2003), but also questions into how humans make adaptive choices and the associated brain regions involved (Montague and Berns, 2002; McClure et al., 2004; Daw et al., 2005, 2011; Daw and Doya, 2006). Recently, electrophysiology (Zaghloul et al., 2009; Patel et al., 2012) and electrochemistry (Kishida et al., 2011) experiments in have been used to investigate the role of dopaminergic neurons and dopamine release in human decision-making.

Among the first investigations into the role human dopamine neurons play in decision-making behavior is Zaghoul and colleagues' recordings of neural activity in human substantia nigra (Zaghloul et al., 2009). The participants for this study were patients undergoing deep brain stimulation electrode implantation for the treatment of Parkinson's disease. During surgery, acutely implanted sharp electrodes recorded neural activity while patients played a gambling task. The authors demonstrated that unexpected gains (but not expected gains) were associated with increases in the firing rate of neurons in the substantia nigra. These results are consistent with dopamine neurons in the substantia nigra of humans tracking a reward prediction error and are consistent with reinforcement learning theories of dopamine neuron activity previously only studied in model organisms. In addition, Patel and colleagues recently measured single unit activity in the nucleus accumbens during a financial decision-making task (Patel et al., 2012). Results from these experiments demonstrated that single unit activity in the nucleus accumbens predicted the choice the participants would express 2 s later; and, the activity in these neurons was reported to encode the difference between the expected and realized outcome, which is also consistent with a prediction error signal.

**Figure 2:**
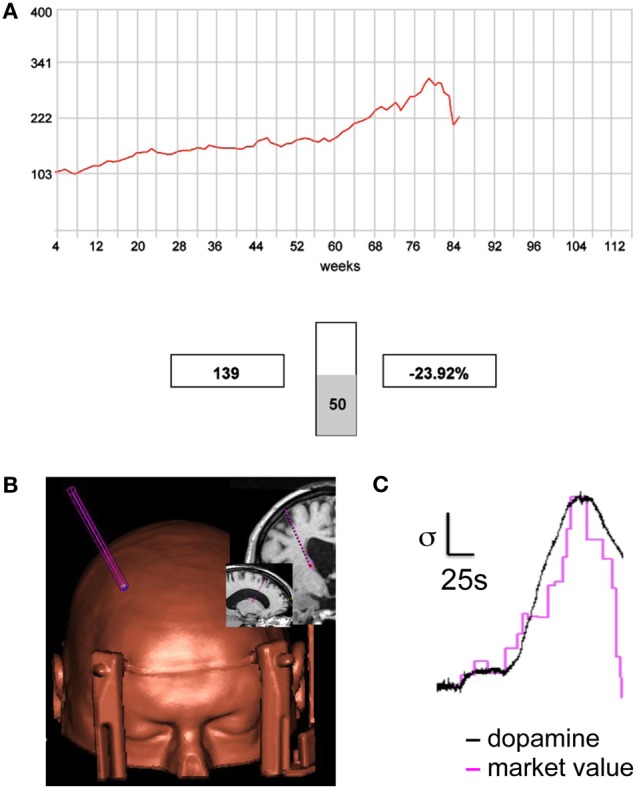
**Sub-second dopamine release during the sequential investment task. (A)** Screen shot from the sequential investment task (Lohrenz et al., 2007; Chiu et al., 2008; Kishida et al., 2011). Participants are provided (1) a trace of the market, (2) the value of their portfolio (shown in this screen shot: “$139”), and (3) the fractional change in the value of their portfolio following their most recent investment decision (shown: “−23.92%”). Participants lodge investment decisions using button boxes that control a visually displayed vertical slider bar. **(B,C)** Fast-scan cyclic voltammetry on a carbon fiber microsensor was used to track dopamine release in the caudate of a human patient performing the sequential investment task. **(B)** A reconstructed image showing the microsensor trajectory and the depth target in the caudate (see MRI image insets). **(C)** Measurements of extracellular dopamine track the market value. Magenta trace: market value as the participant lodges investments (20 decisions shown); Black trace: measured dopamine release in the patient's caudate sampled at 10 Hz. Scale bars (normalized units): vertical bar indicates one standard deviation; horizontal bar indicates 25 s (Kishida et al., 2011).

The implantation of deep-brain-stimulating electrodes for the treatment of Parkinson's disease and a growing number of other neurological disorders is beginning to open the door to invasive neurophysiological measurements in humans. The opportunity to relatively safely record measurements directly from human brains invites the development of new technology to gain further understanding in to human brain function. Along these lines, Kishida and colleagues, adapted carbon fiber microsensors to perform fast-scan cyclic-voltammetry in humans (Kishida et al., 2011). Fast-scan cyclic-voltammetry has been used to measure rapid changes in extracellular dopamine concentration in freely moving rodents (Phillips et al., 2003; Clark et al., 2010). Kishida and colleagues, used this technology for the first time in humans to measure dopamine release in human striatum during a financially incentivized sequential investment game (Figure 2A, adapted from Kishida et al., 2011). This game had been used in fMRI experiments to test the impact different kind of learning signals have on the choices expressed by healthy participants (Lohrenz et al., 2007) and participants addicted to nicotine (Chiu et al., 2008). In these prior experiments participants' choices were predicted by experience-based learning signals or counterfactual fictive learning signals depending on their state of addiction and whether or not they were craving or sated on nicotine (Chiu et al., 2008). These experiments demonstrated that the BOLD response tracking these two learning signals appeared in the caudate and that these signals were (1) important for the expression of adaptive behavior in humans and (2) that the status of being an addict or being sated or unsated has a significant effect on the expression of adaptive behavior.

The experiments executed by Kishida and colleagues used a carbon fiber microsensor placed in striatum of a human participant (Figure 2B, left inset) to measure dopamine release 10 times per second while the patient performed the sequential investment game (Figure 2A). The major importance of this study was the demonstration of new technology to investigate the computational role of dopamine release in a human brain. However, there was also a surprising result in that the dopamine concentration was not shown to track rewards or losses (expected or unexpected) nor did it track learning signals previously demonstrated in the human fMRI experiments with this task. Rather, the dopamine concentration tracked the “stock market” price with very high correlation (Figure 2C). In the context of the reinforcement learning models this may correspond to a signal about the “state” the decision maker was in. This result is an early demonstration of the importance of investigating the neurobiological mechanisms underlying human choice using a variety of measurement tools and theoretic perspectives.

## Conclusion

The problem of “free will” is an old philosophical one. It can be considered ultimately a problem of choice and about the capacity an individual possess in determining the outcome of choice problems. Intertwined with this problem are those, which engage questions about agency. These questions and problems posed in philosophical terms tend to ignore the fact that these questions are about biological agents—humans [with exceptions (Dennett, 2003; Churchland and Churchland, 2006; Suhler and Churchland, 2009)]. Neuroscience, with the development of new technology, is beginning to investigate a wide range of questions concerning human choice behavior. Computationally framed theories will be important to guide these disciplines in order to be concrete about the relationships between hypothetical computations, expressed behavior, and the underlying biology.

Computational reinforcement learning theory and game theory have already made a significant impact in human decision neuroscience. Not only have they framed the problem of choice in quantitative terms, but also they have begun to open new areas of research in domains where human choice is severely restricted as observed in individuals diagnosed with psychiatric disorders. I argue that continuing along these lines will be important of a neuroscience of human choice capacity, or what it means to have anything like “free will.” This would include beginning with a computational definition of “free will.” Huys and Dayan have made a positive step in this direction by addressing one aspect of what it would mean in computational terms to have “control” (Huys and Dayan, 2009); they propose a Bayesian description of what it would mean for an agent to make choices with a causal relationship to the expected outcomes. Interestingly, this work is developed in the context of trying to understand an aspect of major depression and learned helplessness (Huys and Dayan, 2009). Additionally, Dayan and colleagues use a computational reinforcement learning theory framework to explore the possibility of competition between decision-making systems within an agent and demonstrate theoretical results of the impact competing valuation systems may have on the expression of adaptive behavior (Daw et al., 2005; Dayan et al., 2006).

The development of theory without application to, or verification with, empirical investigation is not a scientific endeavor. Indeed, the theoretical frameworks discussed herein have guided investigations into the neurobiological substrates supporting human choice. The primary tool thus far has been fMRI, however, new developments in access to human brains and supporting clinical and investigational technology is providing an unprecedented look into the neurobiology of human choice and agency. Going forward, the biology of willful choice behavior in humans is wide open for investigation and will likely make major advances in our basic understanding of the underlying neurobiology. More practically speaking, these developments will also likely have a major impact on our understanding of human mental illness—conditions where capacity for choice becomes so restrictive that individuals suffer without apparent alternatives. What it means to have “free will” isn't very clear and this concept continues to evolve, but it is clear that humans express choice and have preferences. The quantitative exploration of these concepts has provided a clarifying foundation for theoretical and empirical developments. This same framework promises to allow us to measure human choice capacity and in this context we may come to understand and exercise the freedoms we do possess.

### Conflict of interest statement

The author declares that the research was conducted in the absence of any commercial or financial relationships that could be construed as a potential conflict of interest.
